# Improving participants’ recruitment in dementia-related studies on social media through colloquial language in Lima, Peru

**DOI:** 10.1590/1980-5764-DN-2024-0232

**Published:** 2025-06-02

**Authors:** Belén Custodio, Maria Mora-Pinzon, Rosa Montesinos, Maria Eugenia Godoy, José Carlos Huilca, Katherine Agüero, Nilton Custodio

**Affiliations:** 1 Instituto Peruano de Neurociencias, Research Department, Lima, Peru.; 2 University of Wisconsin-Madison, School of Medicine and Public Health, Division of Geriatrics and Gerontology, Department of Medicine, Madison, USA.; 3 Equilibria, Research Department, Lima, Peru.; 4 Universidad Adolfo Ibáñez, BrainLat, Santiago, Chile.; 5 Universidad de San Andrés, Victoria, Argentina.

**Keywords:** Dementia, Patient Selection, Social Media, Healthcare Disparities, Latin America, Demencia, Selección de Paciente, Medios de Comunicación Sociales, Disparidades en Atención de Salud, América Latina

## Abstract

Recruitment for dementia research remains a persistent challenge, particularly when trying to reach underrepresented groups such as Latin Americans with traditional enrollment strategies.

**Objective:**

In this retrospective case study, we investigate two communication approaches used on social media to recruit participants for a dementia research study in Lima, Peru.

**Methods:**

Initially, recruitment materials featured medical terminology, which was later replaced by colloquial language to make the content more accessible.

**Results:**

According to the results, using simpler and more relatable language led to an increase in participants’ contacts and enrollments. This suggests that culturally tailored communication strategies may be more effective in reaching diverse populations.

**Conclusion:**

The findings highlight the importance of adapting research recruitment efforts to the target community’s cultural and linguistic context to improve engagement in dementia studies.

## RESUMEN.

El reclutamiento para estudios de investigación en demencia continúa siendo un desafío, especialmente al intentar llegar a grupos subrepresentados, como los latinoamericanos, mediante estrategias tradicionales.

**Objetivo:**

Este estudio de caso retrospectivo analiza dos enfoques de comunicación utilizados en redes sociales para reclutar participantes en un estudio de demencia en Lima, Perú.

**Métodos:**

Inicialmente, los materiales de reclutamiento empleaban terminología médica, que luego fue reemplazada por un lenguaje coloquial para hacer el contenido más accesible.

**Resultados:**

Los resultados mostraron que el uso de un lenguaje más sencillo y cercano aumentó el número de contactos y enrolamientos de participantes. Esto sugiere que las estrategias de comunicación culturalmente adaptadas pueden ser más efectivas para alcanzar poblaciones diversas.

**Conclusión:**

Los hallazgos resaltan la importancia de adaptar las estrategias de reclutamiento a la realidad cultural y lingüística de la comunidad a la cual se desea llegar, para mejorar la participación en estudios sobre demencia.

## INTRODUCTION

In 2019, 4.5 million Latin Americans and 107,824 Peruvians were estimated to be living with dementia, and this number is expected to increase by 200% in 2050^1^. A growing body of evidence highlights the underrepresentation of vulnerable Latino populations in Alzheimer’s disease and related dementia (ADRD) research^2^, and how traditional enrollment strategies contribute to this issue. Barriers to recruitment include a lack of clinical trial awareness, study partner requirements, and primary care physicians’ lack of capacity and resources to assess cognition in potential participants^3,4^.

Traditional recruitment strategies have included recruitment via medical professional referral, mail, television, and radio advertisements. While these strategies have been shown to be effective, they have shown varying abilities to reach diverse patient populations^5^. With advancements in technology, new methods are available to promote clinical studies such as online advertisements^6^, social media, phone calls or emails^7^. However, certain cultures, such as the Japanese, might prefer newspapers to television and the Internet, suggesting that newspaper advertisements still play a role in reaching and encouraging older adults to participate in clinical studies^8^.

While there is evidence supporting web-based recruitment for clinical studies in certain areas, such as smoking cessation, mental health, hypertension, and cancer^8^, there is limited research on its application in neurological diseases, with few studies utilizing online recruitment for ADRD trials^3,9^. Authors of a literature review found that while social media is used to recruit individuals from various age groups, 96% of the studies are from high-income countries, indicating a need to document experiences from other contexts, as there are limited guidelines and recommendations for research teams unfamiliar with this emerging recruitment method^10^.

The rising adoption of technology and Internet use among the Peruvian community^11^ presents an opportunity for online recruitment of research participants, with about 69% of Peruvians active on Facebook^12,13^. In Peru, there is limited information on the effectiveness of online recruitment for enrolling older adults in ADRD-related research and the cultural adaptations need to improve participants’ enrollment.

In this retrospective case study, we outline two social media communication strategies employed to recruit participants for the Multi-Partner Consortium to Expand Dementia Research in Latin America (ReD-Lat). We expect that this research will serve as a basis for future outreach and recruitment efforts in ADRD-related research, especially in underrepresented populations.

## METHODS

### The study: ReD-Lat, the Multi-partner Consortium to Expand Dementia Research in Latin America

ReD-Lat is a multisite cross-sectional study aimed at combining genomic, neuroimaging, and behavioral data to improve dementia characterization and identify novel inroads to treat neurodegeneration in diverse populations from Latin America, including controls and patients with Alzheimer’s disease (AD) and Frontotemporal dementia (FTD). Participation in the study involves medical evaluation, blood draw, neuropsychological evaluation, and magnetic resonance imaging.

### Recruitment strategy

Each country in the project designed and implemented its own recruitment strategies based on local needs, without the need for central approval. In Peru, the main strategies include presenting the project at community venues, dissemination in social media, advertisements in healthcare centers and public places, and presenting the project to other doctors for patient referrals. In addition, word-of-mouth effectively works — if a participant being evaluated leaves happy and with a pleasant experience, they are likelier to tell other people about the study.

**Figure 1:**
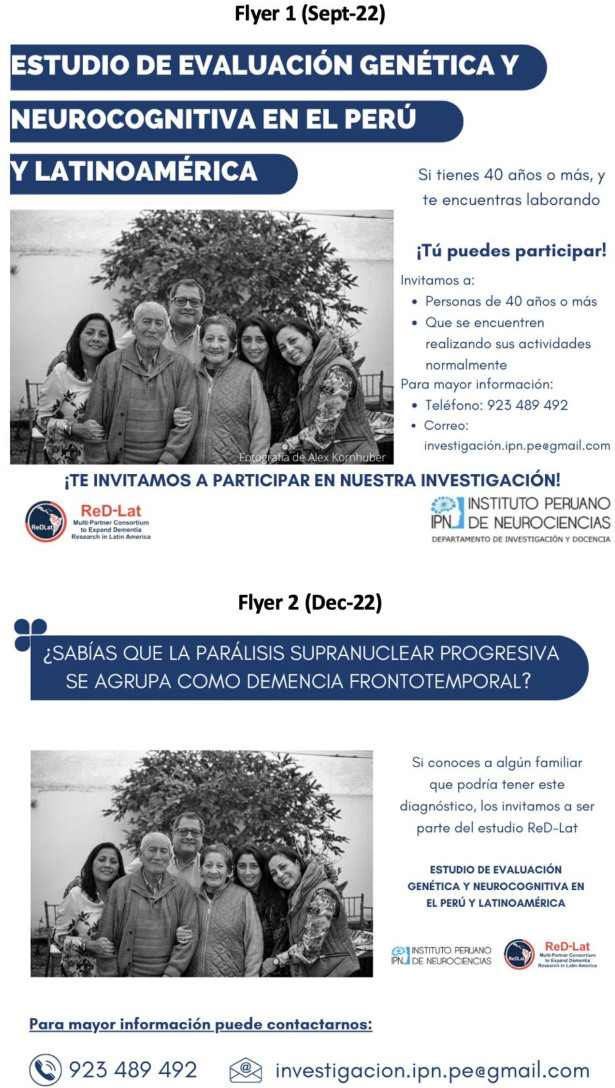
Approach 1: Using medical and common terms in research, Flyers 1 and 2.

**Figure 2:**
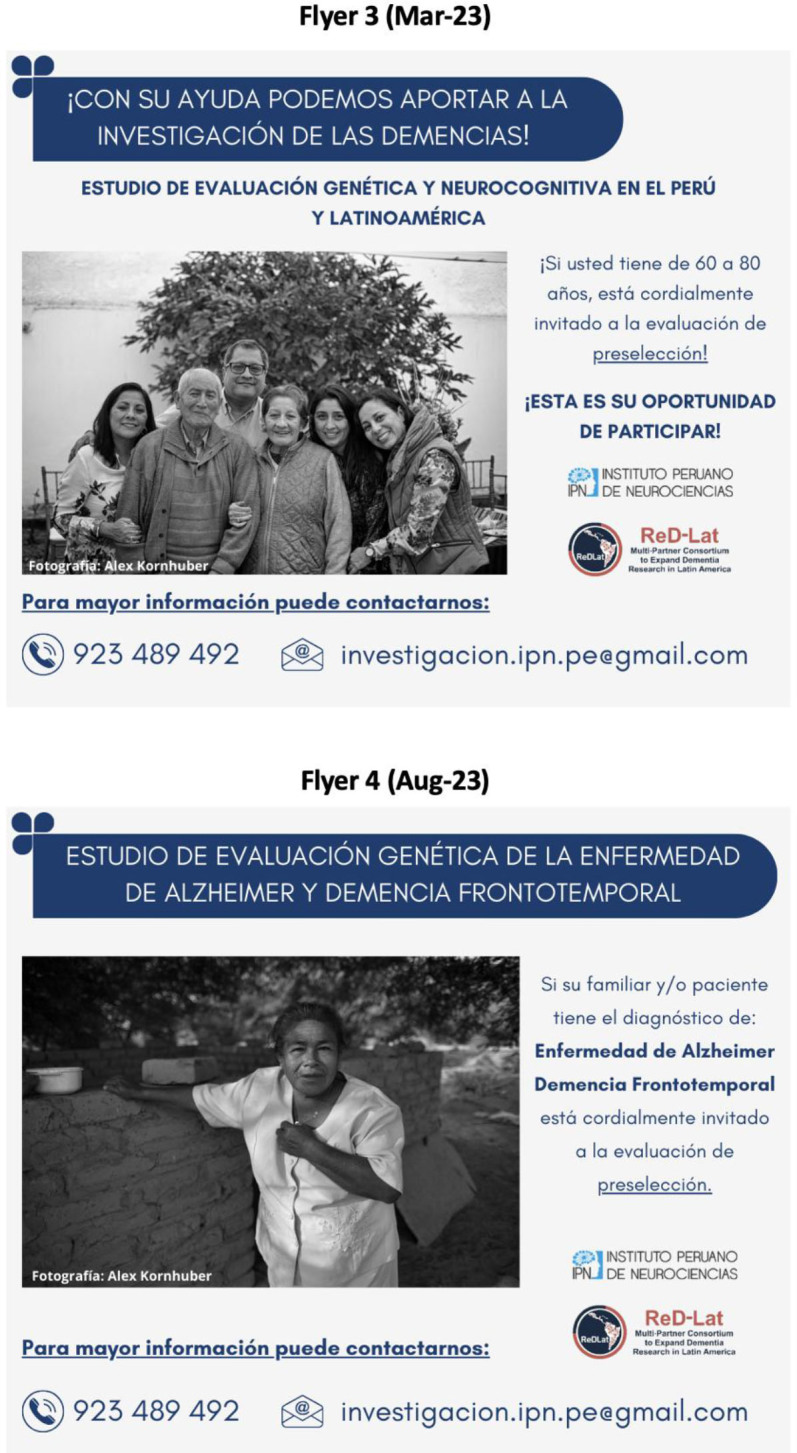
Approach 1: Using medical and common terms in research, Flyers 3 and 4.

The flyers and posts were written in Spanish, although for this article we have provided English translations (Figures 1 and 2). All materials were locally designed and approved for its distribution. Each flyer includes the logo of the project (ReD-Lat) and of the Peruvian Institute of Neurosciences (*Instituto Peruano de Neurociencias* – IPN). Additionally, each material features a photo of Peruvian people, taken by a Peruvian photographer in various regions of the country.

Interested people were directed to contact the research team via the telephone or email provided in the flyer. If they inquired about more information through Facebook comments or private messages, they were given the contact information and offered the option to share their number for a team member to call them. After contacting the study coordinator, participants received more study details and were invited to schedule a screening visit. Eligibility was verified during the visit, and if they agreed to participate, they signed the informed consent form.

### Online recruitment approaches

All posts were shared on Facebook through IPN’s account. To expand the audience, all publications were boosted (Paid Advertisement) targeting people aged 18 to 65 years living in Lima, Peru.

**Figure 3:**
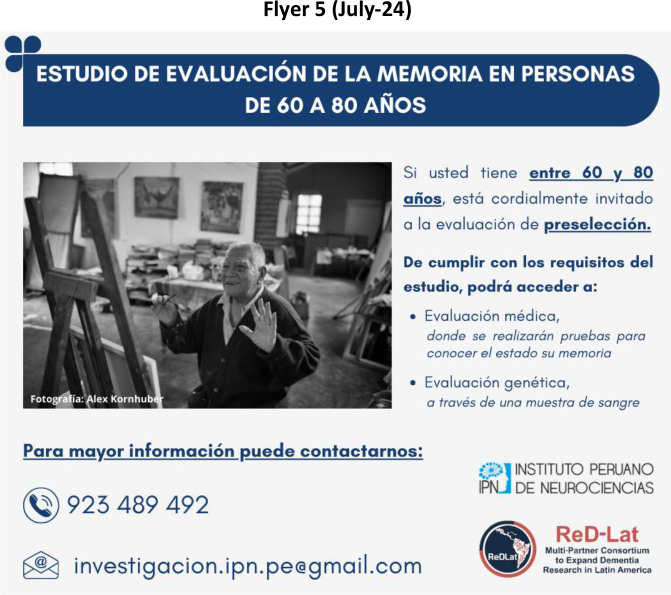
Approach 2: Using colloquial language.

A total of five posts were made, with the first four published approximately every three months, and the last one a year later. Only one flyer was circulated at a time. The characteristics of each flyer, period of distribution, target population, flyer’s title, and main changes from the previous version are shown in Table 1. Overall, two strategies were tested during the study period: Approach 1 – Using medical and common terms in research (Figures 1 and 2), and Approach 2 – Using colloquial language (Figure 3), which refers to shifting from medical terms and disease names that may not be familiar to the general population to more commonly used, everyday language.

**Table 1:** Summary of the flyers and changes over time.

	Date of publication	Target population	Title	Content	Changes from previous version
Flyer 1	September 2022.	Cognitively-healthy participants.	Genetic and neurocognitive assessment study in Peru and Latin America.	Age and “people doing their activities regularly” specified as inclusion criteria. The sentence aimed to recruit control participants without memory issues, but was not explicitly stated in the flyer.	Not applicable.
Flyer 2	December 2022.	Families of patients with FTD.	Do you know that progressive supranuclear palsy is classified as frontotemporal dementia?	The photo remains the same and the following sentence was added: “If you have a family member with this diagnosis, he/she can be part of the ReD-Lat study.”	The contact information was relocated and the font size was increased.
Flyer 3	March 2023.	Cognitively-healthy participants.	With your help, we can contribute to dementia research.	The contact details were positioned and formatted like in the second flyer, and the pi cture remained unchanged. The flyer also mentioned the age range that could schedule a prescreening evaluation.	Engaging title, but keeping the medical terms in the subtitle.
Flyer 4	August 2023.	Families of patients with Alzheimer’s disease or FTD.	Genetic evaluation study of Alzheimer’s disease and frontotemporal dementia.	The flyer indicated that if a family member had these diagnoses, they were invited to the prescreening assessment.	New image (Woman in a garden).
Flyer 5	July 2024.	Cognitively-healthy participants.	Memory evaluation study on people aged 60 to 80 years.	It was mentioned that people aged 60 to 80 years were invited to the prescreening assessment.	New image (Man in an art studio); Avoid Medical Jargon; Includes Benefits of Participation.

FTD, Frontotemporal dementia.

Once Approach 2 was implemented (July 2024), the content also evolved by adding the study benefits, including the text: “By meeting the study criteria, you will be able to have access to:”, followed by an explanation of the benefits for eligible participants: “medical evaluation, including tests to check their memory status, and genetic evaluation through a blood sample.”

### Data collection

Since January 2023, everyone contacting the Research Department for study information has been asked how they heard about the study. This data is recorded in an internal database to track those who requested information, scheduled a screening visit, and those who agreed to participate.

## RESULTS

Since January 2023, a total of 57 people have contacted the research team to request more information, 47 of them scheduled a screening visit (82%), and 32 were enrolled (56% of those interested). In Table 2 we show the distribution of Facebook followers at the time of each post, individuals reached and enrolled based on advertisement that was active at the time, showing a progressive increase in the enrollment rate.

**Table 2:** Summary of the results of each Facebook post.

	Facebook followers at the time of each post	Number of people reached per post (Reach)	Number of people that contacted the study team* (n=57)	Number of evaluations (screening) (% of those that contacted the team)	Number of individuals enrolled (% of those that completed the evaluation)
Flyer 1	18,605	7,498	N/A	N/A	N/A
Flyer 2	18,954	9,269	N/A	N/A	N/A
Flyer 3	19,218	10,084	13	10 (77)	5 (50)
Flyer 4	19,904	13,651	15	13 (87)	9 (69)
Flyer 5	21,306	29,636	29	24 (83)	18 (75)

Notes: *People who mentioned seeing the advertisement on social media; N/A, not applicable.

Reasons for not enrolling varied between the flyers. In the third flyer, a total of eight people were not enrolled in the study for the following reasons: five (62% of those not enrolled) did not meet the inclusion criteria, one lived outside the country, one was interested in another study, and contact was lost with the last person. For the fourth publication, six people did not reach the enrollment phase, as two (33%) did not meet the inclusion criteria, two declined to participate in the study, and contact was lost with two other participants. For the last published flyer, where medical and research-related terms were avoided, six (54%) people did not meet the inclusion criteria, and five declined to participate.

## DISCUSSION

In this retrospective case study, we investigated the impact of two communication approaches on participant recruitment for an ADRD-related research study. The first used formal medical terms, while the second employed colloquial language to make the content more accessible. The shift to simpler language led to greater interest, suggesting that accessible communication positively impacts participation. Our findings align with other publications whose authors showed that using vibrant images and simple messages on social media improved recruitment of men for a physical activity intervention^14^. Other researchers highlighted that selecting appropriate text and images is essential for effectively using social media’s reach in recruiting participants for an online mental health study^15^. Our results also align with results from a mixed-methods study in Wisconsin, where Latino community members highlighted a preference for posts that combined photos of real individuals with text, rather than text-heavy posts^16^.

Using appropriate language also promotes the development of trust between the participant and the research team, as explained by Moreno-John et al.; they point out that all the research should be culturally adapted to the community being addressed, this includes not only materials, but also the whole research team, which helps to overcome any language barriers that may exist^17^. A successful example of this strategy is the study carried out by Ashford et al. — over 2.25 years, researchers developed a culturally-tailored digital aid with Latinos, which included a culturally-tailored enrollment website and Facebook ads created through a community-engaged research approach to enroll 5 thousand older Latino adults from California into the Brain Health Registries; this approach resulted in the recruitment of 3,603 older adults in less than half the estimated time (71% of the enrollment goal)^9^. Other studies have also showcased that trust and credibility of a project are enhanced by including the project’s institutional logo^14^, which reflects the importance of our posts displaying the ReD-Lat and IPN logos, while also displaying them on the flyers shared on IPN’s Facebook page.

Although social media is indeed a relatively new communication channel and a large part of the users are young people, in our case, we used Facebook to recruit older adult participants, as this is the channel consistently preferred by older people^18,19^. Although Facebook was our main way to disseminate the information of the study, our success in scheduling the medical evaluations and recruiting individuals shows the importance of having the first contact offline to build the participant’s trust in the team/research. This finding is consistent with previous studies whose authors evidenced that personal contact is relevant to generating the bond of trust between the participant and the healthcare professional, also aligning with some of the core cultural values of personalism, which means a preference for communication and warm contacts as ways to build trust^20-22^.

Regarding the number of followers, while the IPN’s Facebook page experienced an increase, which could potentially impact the reach of posts, we do not consider this to be the primary explanation for the improvement in the enrollment rate. This is because the average organic reach of Facebook posts declined from 7.7% in 2019 to 2.6% in 2024^23,24^, meaning that fewer users are seeing posts over time. As a result, paid advertisements have become necessary, as they are targeted towards individuals with similar interests. Another important factor to consider is how Facebook’s algorithm operates, which consists of a complex set of rules that enables the platform to function in a personalized way, prioritizing content that each individual finds engaging^25^.

Despite the valuable insights, there are limitations to consider, including the lack of continuous posting. Some posts were made every three months, with gaps of over six months between related posts, which could have an impact on the reach of the messages and subsequently limited the number of people that decided to call to learn more about the study. According to research from marketing and business fields, an individual needs to see a message at least three times before deciding to take action, and an ideal strategy is having posts once or twice a week to create recognition in the audience^26^. Another consideration is that the flyers were posted on the IPN’s Facebook account, a private neurology clinic that shares content related to both research and clinical care. This may have led to previous patients or individuals familiar with the clinic seeing the posts, which could increase their confidence in contacting the provided numbers.

Further studies should explore the effectiveness of digital recruitment methods in reaching more representative patient populations. Furthermore, considering that people using social media might not be representative of the whole population, researchers should use other recruitment strategies and platforms to reach out to individuals, and it will be necessary to use other comparison methods to assess the impact on each strategy in reaching out to specific demographics.

In conclusion, social media advertising reaches a larger overall population and can be effective in diversifying the study population. In this short report, we found that Peruvian Facebook users had increased contact with the research unit and were more likely to participate in the screening and subsequent enrollment process after a change in communication to a simpler language. According to our results, a direct and friendly (colloquial) message to the target audience of the research will remain an important point to be taken into account across recruitment strategies. These results can provide important suggestions for future promotion for recruiting participants to clinical ADRD-related studies in Peru.

## Data Availability

The datasets generated and/or analyzed during the current study are available from the corresponding author upon reasonable request.
